# Perineal sound recording for diagnosis of bladder outlet obstruction

**DOI:** 10.4103/0970-1591.45545

**Published:** 2009

**Authors:** Tim Idzenga, Johan J. M. Pel, Ron van Mastrigt

**Affiliations:** Department of Urology, Sector Furore, Erasmus MC, Rotterdam, The Netherlands; 1 Department of Neuroscience, Vestibular Group, Erasmus MC, Rotterdam, The Netherlands

**Keywords:** Bladder outlet obstruction, non-invasive urodynamics, perineal sound recording

## Abstract

**Objectives:**

Elderly men are prone to developing lower urinary tract symptoms (LUTS) possibly caused by bladder outlet obstruction (BOO). The most frequently used method to diagnose this condition is an invasive and time-consuming pressure-flow study. We are developing a novel non-invasive method to diagnose BOO in men with LUTS based on perineal sound recording.

**Methods:**

A biophysical model urethra was made from polyvinyl alcohol (PVA) cryogel with viscoelastic properties comparable to those of the male pig urethra. To this model different degrees of obstruction were applied and sound was recorded at different positions downstream of the obstruction. In a study in 16 healthy male volunteers the variability and repeatability of perineal sound recording was tested.

**Results:**

In the model three parameters, derived from the frequency spectrum of the recorded sound (i.e., weighted average frequency, standard deviation and skewness) are uniquely related to the degree of obstruction (linear regression, *P*<0.001). The variability of perineal sound recording in healthy male volunteers was found to be smaller within volunteers than between volunteers (Kruskal-Wallis, *P*<0.001) and the repeatability was comparable to that of the maximum flow rate.

**Conclusions:**

We conclude that perineal sound recordings are significantly different between volunteers. In combination with the unique relations found in the model-experiments these results increase the probability that perineal sound recording can be used as a simple and cheap non-invasive method to diagnose BOO. Clinical testing of this method is therefore strongly indicated.

## INTRODUCTION

**Figure 1:**
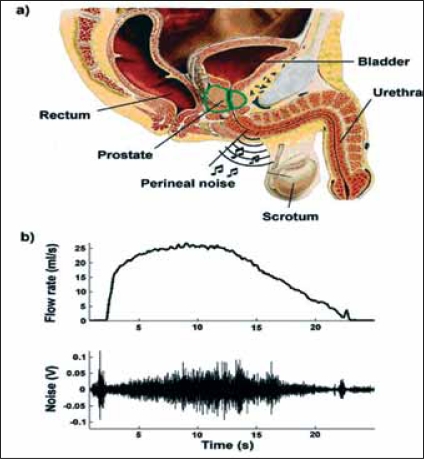
Cross-section of the lower urinary tract (a) and an example of a perineal sound recording in a volunteer (b)

Elderly men are prone to developing lower urinary tract symptoms (LUTS), such as a weak urinary stream, frequent (nocturnal) voiding and incomplete emptying of the bladder. The two most probable causes for these symptoms are a weakly contracting bladder muscle or increased urethral resistance (bladder outlet obstruction; BOO). In most cases, the voiding symptoms are caused by BOO, which is, in turn, frequently caused by benign prostatic enlargement (BPE). The most frequently used method to diagnose this condition in men with voiding symptoms is an invasive pressure-flow study.[1] This testing method however is time-consuming, expensive, and patient-unfriendly. Therefore it is often decided to perform a transurethral resection of the prostate (TURP) based on only a flow rate measurement. A low flow rate can also indicate a weakly contracting bladder and in that case, TURP does not improve the symptoms. To make urodynamic testing of patients with LUTS easier several alternative non-invasive methods have been developed.[2–5] Some of these methods require interruption/manipulation of the voiding[2–4] or expensive equipment.[5] We are developing a novel non-invasive method to diagnose BOO. This method is based on sound, recorded during voiding, using a simple microphone pressed against the perineum [Figure 1]. It is hypothesized that this recorded sound is related to the degree of obstruction.

## METHODS (1)

### Hypothesis

The hypothesis that sound, recorded during voiding, could be used as a diagnostic method originated in the mid-sixties with a voiding audiograph.[6] It was thought that the splashing sound of urine voided in a cup, recorded using a microphone and corrected for the voided volume, was a measure for bladder pressure. One decade later, the microphone was moved from the cup to the body at the level of the perineum and the sound produced in the urethra was recorded simultaneously with the flow rate.[7] It was hypothesized that urinary flow is turbulent at the bladder neck. These turbulences cause pressure fluctuations on the wall of the urethra that can be recorded as sound transmitted via the urethra to the skin.[8] In silicone[9] or latex[10] test tubes it was shown that pressure fluctuations are indeed induced by flow and that the power spectrum of the recorded sound depended among others on the degree of obstruction applied. However, it was not known how the degree of obstruction could be derived from the power spectrum.

In a review on bio-acoustic signals it was noted that phonoangiographic methods, developed in cardiology, might be non-invasive and inexpensive, but that clinical application of these methods has been overtaken by large-scale application of ultrasound Doppler.[11] Nevertheless, a method based on perineal sound recording would make diagnosis of BOO cheap and simple. Therefore, over the years attempts have been made to relate sound recordings from biological fluids (e.g., blood, urine) to the obstruction passed (e.g., stenosis, prostatic obstruction). Despite encouraging results, a non-invasive measurement device is not yet available for urology. One limiting factor is that the test tubes used in earlier experiments are rather stiff compared to the more flexible and distensible blood vessel or urethra, so that results obtained in these test tubes cannot be translated to the clinical situation. Recently, it was reported that flexible and distensible tubes can be made from a 10% aqueous solution of polyvinyl alcohol (PVA) cryogel.[12] The number of freeze-thaw cycles, the rate of freezing/thawing and the concentration of the PVA control the viscoelastic properties of this gel.

To test the hypothesis that sound recorded downstream of an obstruction is related to the degree of the obstruction, we constructed PVA models with varying viscoelastic properties.[13] To these models different degrees of obstruction were applied and the position of the recording microphone relative to the obstruction was varied. The average amplitude (*A*) of the recorded sound and the weighted average frequency (*f_c_*) of its frequency spectrum were found to depend significantly on the degree of obstruction.

### Results (1)

Both parameters appeared to increase with increasing degree of obstruction. This result supported the hypothesis that a non-invasive diagnostic method for BOO might be based on perineal sound recording. Both and depended significantly on the microphone position and on the viscoelastic properties of the PVA-model. This made it necessary to develop a model of the urethra with viscoelastic properties as close as possible to those of the human male urethra.

## METHODS (2)

### A biophysical model of the male urethra

To establish which model urethra best represents the human male urethra the viscoelastic properties of different models were measured. The number of freeze-thaw cycles of the cryogel, the profile of the flow channel in the model and the number of cross-sectional layers were varied. The rate of freezing/thawing and the concentration of the PVA cryogel were kept constant in all models. To select the model urethra with viscoelastic properties comparable to those of the human male urethra the male pig urethra was used as a reference because of the similarities between pig and human physiology. We measured the viscoelastic properties using the method developed by Coolsaet *et al*.[14] and adapted it for use in cylindrical structures. This method involves the stepwise application of strain to the urethral wall by rapidly injecting known volumes of water into the sealed urethra and recording the pressure response.

**Figure 2:**
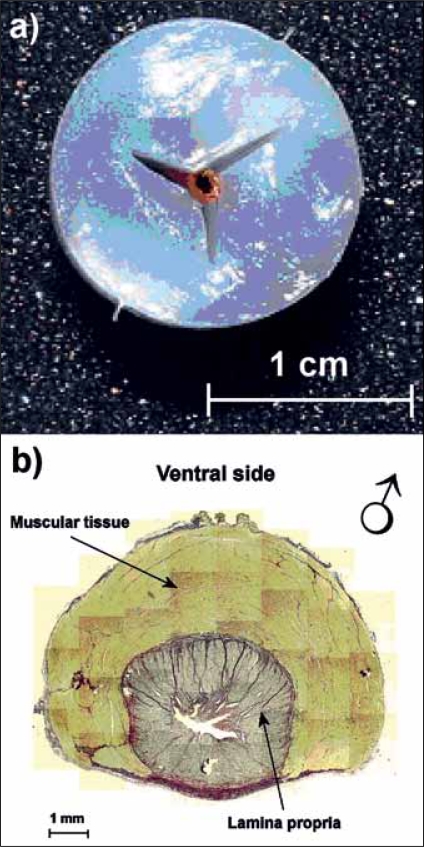
Cross-sections of a PVA model urethra with a Y-shaped opening (a) and of the proximal part of the male pig urethra (b). We stained elastin, collagen (both dark grey) and muscle tissue (light grey) using elastin von Gieson staining. The lamina propria is surrounded by muscular tissue with a horseshoe-like shape. Panel b was reproduced from Urodinamica 16, 310–320, 2006, with kind permission from Editrice Kurtis.

12 different model urethras were constructed by pouring a 10% aqueous solution of PVA into a cylindrical mould. After 6 h of rest at room temperature (21°C), the mould was stored in a freezer at -20°C. It remained in the freezer for 14 h and was then stored at room temperature again for 10 h. This completed one freeze-thaw cycle. To create a channel allowing flow through the models, either a strip with a Y-profile (with legs 5 mm wide, Figure 2a) or a circular rod (diameter 4 mm) was placed along the central axis of the mould. The Y-profile reflected the lumen of a male pig urethra without flow [Figure 2b] and the circular profile reflected the expected profile of a male pig urethra during flow. From 8 male pigs sacrificed at the Department of Experimental Cardiology (Erasmus MC, Rotterdam, The Netherlands) bladder/urethra preparations were made. The part of the urethra closest to the bladder (proximal part) and distal to the prostate was selected. The average (±1 SD) length of the sections was 65 × 16 mm. An example of a cross-section is shown in Figure 2b.

The models were placed in a water-filled container (approximately 1 cm below the water-surface) at room temperature and the pig urethras were placed in the same container (approximately 1 cm below the solution-surface) but filled with a cold (10°C) modified Krebs solution (118 mM NaCl; 4.7 mM KCl; 25 mM NaHCO_3_; 1.2 mM KH_2_ PO_4_; 1.8 mM CaCl_2_; 1.2 mM MgSO_4_, and 11 mM glucose aerated with 95% O_2_ and 5% CO_2_ at pH 7.4). One side of the model or pig urethra was connected to a 5 ml syringe to inject a known volume of water in a very short time. The pressure response was recorded using a disposable pressure transducer connected to the other side of the urethra. Increasing volumes were injected, starting at 0.2 ml with increments of 0.2 ml to a maximum of 5 ml, or to the volume at which the range of the pressure transducer (∼250 cm H_2_O) was exceeded. After each measurement the injected water was extracted. The transducer signal was sampled at a frequency of 1000 Hz using an AD-converter (PCL-818, Advantech^®^) and stored in a PC for further analysis using custom-written programs in Matlab^®^. For an approximate calculation of the viscoelastic properties of the model and pig urethras from the recorded pressure response a mechanical model of the urethral wall was constructed, consisting of springs, dashpots, and a mass.[15] The simplified analytical solution for the step-response of this model was fitted to 1000 samples (equivalent of 1 s) of the pressure signal.

### Results (2)

One of the results found in the model urethras was that stress increased more rapidly as a function of strain with increasing number of freeze-thaw cycles. This implies increased stiffness, which was also found by Chu and Rutt.[12] Furthermore, two time-constants (τ_1,2_) were found for the stress-relaxation in the model and pig urethral wall. In the male pig urethra, these two time-constants differed by a factor of ∼10 from each other, which supports the assumption that two independent relaxation processes are activated after stepwise application of strain. Statistical analysis (Mann-Whitney U-test) was used to compare the viscoelastic properties measured in model urethras to those measured in male pig urethras. Based on this comparison we concluded that a model urethra with a Y-shaped flow channel that was uniformly freeze-thawed three times showed best agreement with the male pig urethra. Therefore, this model was selected to study the relation between recorded sound and the degree of obstruction.

## METHODS (3)

### Recorded sound related to the degree of obstruction?

The flexible and extensible biophysical model which we developed[15] was placed in a container filled with water to prevent evaporation. Different degrees of obstruction were imposed by inflating a blood pressure cuff around the model and sound was recorded at different positions downstream of the obstruction.[16] One side of the model was connected to a water column with regulated water level and the other side to an outflow tube, which drained into a rotating-disk flow meter. The blood pressure cuff formed a distensible obstruction of the model urethra, similar to an elastic prostate, and was connected to a second water column. The level in the first water column (bladder pressure) was varied between 60 and 180 cm and in the second column (cuff) between 20 and 180 cm (both in steps of 20 cm) resulting in 63 possible combinations. Only in 35 combinations of the two column levels a flow rate higher than 2 ml/s was realized. The degree of obstruction was quantified by the bladder outlet obstruction index (BOOI):[17]

[1] $$\mathrm{BOOI}=P_{\det}-2.Q_{\max},$$

with *P*_det_ (cmH_2_ O) being the detrusor pressure at maximum urinary flow rate, modeled by the pressure-drop over the model urethra, and Q_max_ (ml/s) the maximum flow rate through it. According to the international golden standard[1] an unobstructed urethra is defined by BOOI <20 and an obstructed urethra by BOOI >40. For 20 < BOOI < 40 the pressure-flow study is equivocal. The 35 combinations of column levels resulted in BOOI values between -17 and +118 with a mean of 51 and a standard deviation of 32.

From each sound recording, the average amplitude and the frequency spectrum P(f) were calculated using fast Fourier transform. From each frequency, spectrum three parameters were derived: the weighted average frequency *f_c_* (Hz), the standard deviation σ (Hz), and the skewness γ_1_ (-). The parameters derived from the recorded sound might depend on: the bladder pressure, the flow rate, and the cuff pressure. In the model urethra these three variables are related, any one of the three can be calculated from the other two. Therefore each parameter can be described as a function of two independent variables, e.g., bladder pressure and flow rate.

## Results (3)

**Figure 3:**
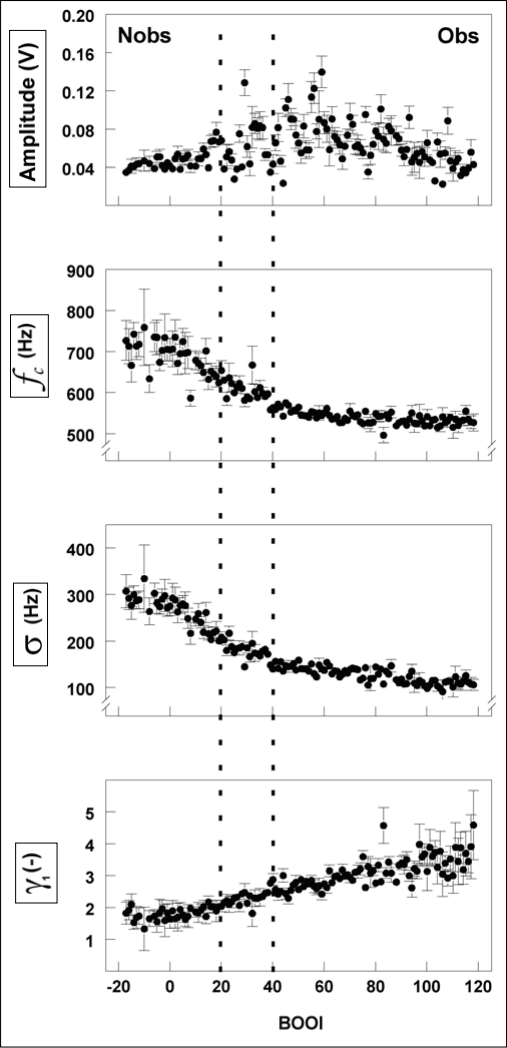
Four parameters, i.e., the average amplitude of the sound and the weighted average frequency (*f_c_*), standard deviation (σ), and skewness (γ_1_) of its frequency spectrum, derived from a sound recording in a PVA model are plotted as a function of the degree of bladder outlet obstruction (BOOI). At each value of BOOI, the mean and standard error of the mean of 5 recordings at 12 distances downstream of the obstruction are shown. The critical BOOI-values are indicated by the dotted lines, unobstructed (Nobs) is defined by BOOI <20 and obstructed (Obs) is defined by BOOI >40 (1, 17). This figure is reproduced from IEEE Transactions on Biomedical Engineering, doi: 10.1109/TBME.2008.919131, © 2008 IEEE.

All three parameters were uniquely related to the degree of obstruction (Linear Regression coefficient: -0.568, -0.653 and 0.466 respectively, *P*<0.001). These relations are presented in Figure 3. The parameters *f_c_* and σ decreased with increasing degree of obstruction and γ_1_ increased. For all three parameters the mean was found to be significantly different between recordings from obstructed and unobstructed models (Student's t-test, *P*<0.001). A decrease in *f_c_* means a shift of the center of gravity of the frequency spectrum to lower frequencies and a decrease in σ means that the power is more concentrated around this center of gravity. Both indicate that increasing the degree of obstruction adds relatively more power to the lower section of the frequency spectrum, caused by the turbulence in the urinary flow.

The results found in this study are based on experimental values in a biophysical model of the urethra. In humans, anatomical aspects of the obstruction and the lower urinary tract may vary between individuals and possibly affect the sound recording. Therefore, further testing of perineal sound recording in male volunteers and patients is needed to establish the diagnostic value of this method.

## METHODS (4)

### Perineal sound recording in male volunteers

Based on the findings in our model-experiments a measurement setup for perineal sound recording in healthy male volunteers and patients was developed. 16 volunteers were recruited to void at least ten times at normal desire.[18] They filled out an IPSS-form as a measure of LUTS and voided in a standing position with a microphone pressed to the perineum directly behind the scrotum using a modified jockstrap. The microphone consisted of a modified dual head stethoscope (MDF Instruments Europe, Copenhagen, Denmark) with the earpiece replaced by a piezoceramic transducer. The voided volume was measured with an external weight transducer. Differentiation of the voided volume with respect to time resulted in the urinary flow rate.

**Figure 4:**
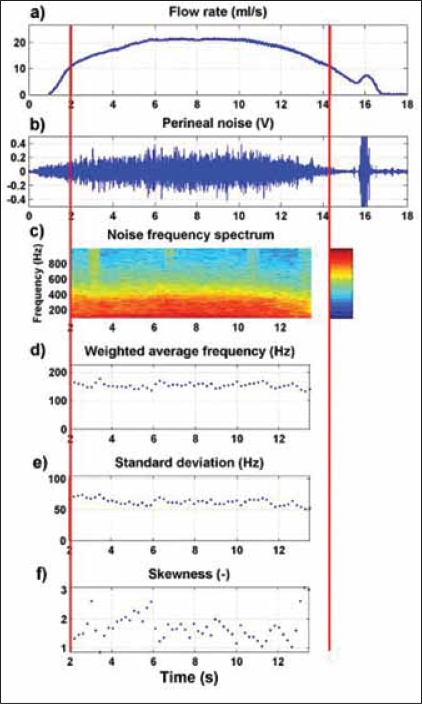
Example of a perineal sound recording in a male volunteer (reproduced from Neurourol. Urodyn., with kind permission of John Wiley and Sons, Inc.). In the example, the flow rate (a), perineal sound (b), and the frequency spectrum of the perineal noise (c) are plotted as a function of time. For each frequency (on the vertical axis) in the sound frequency spectrum red represents a high power and blue represents a low power. In the bottom, three panels the weighted average frequency (d), the standard deviation (e) and the skewness (f), derived from the sound frequency spectrum, are presented as a function of time.

From each sound recording, the part with a flow rate higher than 50 percent of the maximum was selected [see Figure 4]. For this section, the frequency spectrum was calculated between 100 and 1000 Hz in a time-window of ∼0.8 s using fast Fourier transform. The time-window was than shifted ∼0.2 s and the calculation was repeated. This procedure was repeated until the end of the recorded signal was reached. Each frequency spectrum was characterized by the three parameters found in the model-experiments.[16] For each recording subsequently the median value of each parameter was calculated. Also the maximum of the flow rate signal (Q_max_) was assessed.

### Results (4)

**Figure 5:**
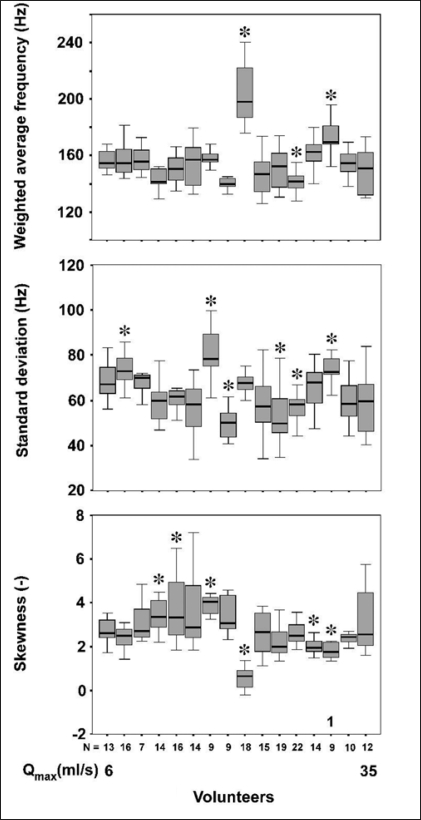
Box-whisker plot of the three parameters, derived from the sound frequency spectra of each volunteer (reproduced from Neurourol. Urodyn., with kind permission of John Wiley and Sons, Inc.). The box represents the median value and the 25th and 75th percentile and the whiskers represent the highest and lowest value. Volunteers that were significantly different from three or more other volunteers (Dunn's test, *P*< 0.05) are marked with an asterisk. The number 1 identifies a volunteer that was significantly at the top end of the weighted average frequency and the standard deviation scale and at the bottom end of the skewness scale.

The variability of all parameters was significantly smaller within volunteers than that between volunteers (Kruskal-Wallis, *P*<0.001). Therefore, for each parameter at least one volunteer had results that were significantly different from the other volunteers. The three parameters are plotted as box-whisker plots in Figure 5 with the volunteers sorted by their mean maximum flow rate (ranging from 6 to 35 ml/s). The volunteers that were significantly different from three or more other volunteers (Dunn's test, *P*<0.05) are indicated with an asterisk. Four volunteers were significantly different in more than one parameter.

To derive a measure of repeatability we have drawn random pairs of recordings from each volunteer data set, with replacement. None of the pairs were identical. The three parameters and the maximum flow rate were measured on different scales. Therefore, for comparison with the repeatability of the maximum flow rate we calculated a slightly modified version of the normalized repeatability statistic used by van Mastrigt and Chung.[19] For each pair of recordings we calculated the difference and the mean of the values of the three parameters and the maximum flow rates. The normalized statistic was calculated as the standard deviation of the differences, divided by the range of the means. The repeatability measures of the three parameters were not significantly different from that of the maximum flow rate (Wilcoxon's signed rank test, *P*=0.438, *P*=0.163 and *P*=0.756, respectively). The variation between the volunteers is most likely a result of physiological variation, e.g. differences in structure and function of the lower urinary tract. The sound recordings in this study are similar to the examples presented by Koiso *et al*.[20] and Teriö.[9] The values for the weighted average frequency given by Teriö for the one volunteer in his study (112 and 129 Hz) are comparable to the values we found in our volunteer population (125–240 Hz). However, the values given for the patients (170–454 Hz) are higher than those in our population. Even though the values are calculated from a slightly different part of the sound frequency spectrum this hints at a possible way to discriminate healthy volunteers from patients.

We calculated the correlation between parameters using the non-parametric Spearman's correlation coefficient. The weighted average frequency had a good correlation with the standard deviation (Spearman's rho = 0.720, *P*< 0.05) and with the skewness (Spearman's rho = -0.699, *P*<0.05). The standard deviation had a weak correlation with the skewness (Spearman's rho = -0.299, *P*<0.05). The correlations between the parameters indicate that when a volunteer is on the top end of one scale he is much likely to be on the top or bottom end of another scale. For one volunteer it was shown that he was significantly (Dunn's test, *P*<0.05, Figure 5) at the described ends of the scales. This was as expected from the earlier published model experiments[16] [Figure 4].

## CONCLUSION

Based on these findings we conclude that perineal sound recording gives repeatable results in healthy male volunteers and that the results are significantly different from volunteers. In combination with the unique relation between recorded sound and the degree of obstruction we found in the model-experiments, these results increase the probability that perineal sound recording can be used as a very simple and cheap method to non-invasively diagnose BOO. Clinical testing of this method is therefore strongly indicated.
